# Comparison of two different contrast sensitivity devices in young adults with normal visual acuity with or without refractive surgery

**DOI:** 10.1038/s41598-022-16855-3

**Published:** 2022-07-28

**Authors:** Hyunjean Jung, Sung Uk Han, Sangyeop Kim, Hyunmin Ahn, Ikhyun Jun, Hyung Keun Lee, Kyoung Yul Seo, Tae-im Kim

**Affiliations:** 1 Department of Ophthalmology, Institute of Vision Research, Yonsei University College of Medicine, 50-1 Yonsei-ro, Seodaemun-gu, Seoul, 03722 Republic of Korea; 2 Department of Ophthalmology, Corneal Dystrophy Research Institute, Severance Hospital, Yonsei University, Seoul, Republic of Korea

**Keywords:** Refractive errors, Vision disorders

## Abstract

This study investigated the reliability and correlation of two contrast sensitivity test (CST) devices in young adults with normal visual acuity, with or without refractive surgery. 57 patients aged 20–39 years who received both manual (OPTEC-6500) and automated CST (CGT-2000) examinations from June 19 to July 24, 2021 were retrospectively enrolled. Patients with corrected visual acuity under 20/20 or history of ocular surgery other than refractive surgery were excluded. 82 eyes of 41 patients (40 eyes with and 42 without history of refractive surgery) were enrolled. Mean time taken to complete each examination was 396.4 ± 20.4 and 286.8 ± 2.3 s using manual and automated CST, respectively (P < 0.001). Patients who underwent refractive surgery had significantly decreased area under the log contrast sensitivity formula (AULCSF) in mesopic compared with photopic conditions in automated CST examinations (AULCSF difference 0.415 vs. 0.323 in patients with and without refractive surgery, P < 0.001), but there was no significant difference in manual CST examinations. Patients who reported decreased subjective night vision had significantly decreased AULCSF in automated CST examinations, but there was no significant difference in manual CST examinations. Compared with manual CST, automated CST was quicker and correlated well with decrease in subjective night vision.

## Introduction

Visual acuity is a commonly used method to check visual power, usually using the Snellen chart. It uses targets of very high contrast and measures spatial-resolving ability. However, Snellen acuity is a measure of only visual quantity and provides limited information about functional vision^1^. On the other hand, contrast sensitivity quantifies the lightness or darkness needed to identify a target against its background^2^. Measuring contrast sensitivity is just as important as visual acuity as it reflects the quality of vision and in many cases declines earlier, while visual acuity remains normal (6/6 or better)^2^. Contrast sensitivity plays a role in many aspects of vision, specifically motion detection, visual field, pattern recognition, dark adaptation, and visual acuity^1,3^, and affects patients’ daily lives.

Many conditions, including age^4^, myopia^5^, higher-order aberrations^6^ and neurologic degeneration including parkinsonism^7^ affect contrast sensitivity, just before any change in visual acuity is detected. Contrast sensitivity changes even in the early stages of cataract^8^, age-related macular degeneration^9^, open angle glaucoma^3^, without necessarily affecting the visual acuity from such an early stage. Therefore, contrast sensitivity tests (CSTs) may help clinicians to understand a patient’s impairment and complaints in those with normal visual acuity^1,10^.

Laser in situ keratomileusis (LASIK) and laser-assisted sub-epithelial keratectomy (LASEK) have gained widespread popularity as a method to correct refractive errors. Although refractive surgery can reduce refractive errors and improve uncorrected visual acuity, previous studies have shown that it can degrade the quality of vision, resulting in reports of reduced night vision clarity^11^ and contrast sensitivity^12^ yet some studies argue that this decline recovers within 3 or 6 months postoperative^13^. Therefore, contrast sensitivity plays an important role in describing the visual function of patients post refractive surgery. However, only a few previous studies have focused on investigating the long-term course of contrast sensitivity after refractive surgery^14^, and to our knowledge, there has been no previous study to compare two devices in patients post refractive surgery.

The first CST devices used wall charts such as the Pelli-Robson chart, which measures contrast sensitivity using a single large letter size (20/60 optotype), with contrast varying across groups of letters^15^. However, certain problems are inherent in paper charts. First, the charts fade over time, making the results less accurate. The lighting environment also affects the results. The chart must be illuminated, but without calibrating the room, ambient light from windows and neighboring exam lanes can alter the results of the test^16^.

These limitations led to the introduction of built-in charts, including the OPTEC-6500 (Stereo Optical, Chicago, IL), in which lighting, distance, and glare are standardized. These are manual CST devices that use pattern fringe stimuli that are presented manually and whose orientation must be identified manually.

The CGT-2000 (Takagi Seiko Co., Ltd., Nagano-Ken, Japan) is an “automated” CST device, where lighting, distance, and glare are standardized as in other built-in CST devices but differs in that it is fully automatic. The presentation of the stimulus lasts for a certain period (0.2, 0.4, 0.8 or 1.6 s according to the examiner’s preference), as in the stimuli of automatic visual field perimetry devices, and its measurement is carried out using an automatic threshold recognition strategy. The automated CST device is a patient-driven, standardized test that can easily maintain examination conditions under control and eliminate technician bias^17^. If they show comparable repeatability and reliability, automated CST devices can be useful instruments to measure contrast sensitivity quickly and easily for patients.

In this study, we assessed the repeatability and correlation of a manual CST device (OPTEC-6500) and an automated CST device (CGT-2000) in healthy young adults with normal visual acuity with or without a history of myopic refractive surgery (LASIK or LASEK).

## Methods

### Study population

**Figure 1:**
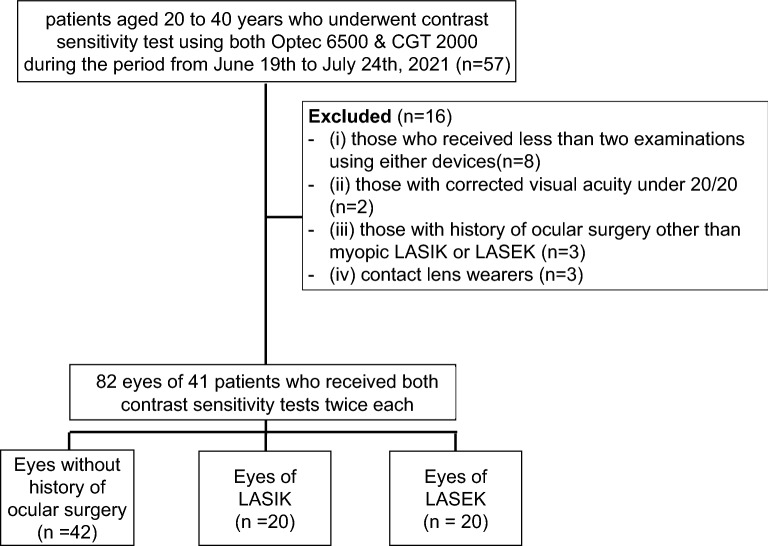
Flowchart of the study population. LASIK, laser-assisted in situ keratomileusis; LASEK, laser-assisted sub-epithelial keratectomy; s/p, status post.

This was a retrospective study of patients aged 20 to 39 years who underwent contrast sensitivity tests using both manual and automated CSTs from June 19 to July 24, 2021 at Severance Hospital, Yonsei University College of Medicine (n = 57). Subjects with the following were excluded: (i) those who received less than two examinations using either device (n = 8); (ii) those with corrected distance visual acuity under 20/20 (n = 2); (iii) those with a history of ocular surgery other than myopic LASIK or LASEK (n = 3); and (iv) contact lens wearers (n = 3) (Fig. 1).

All participants responded to a survey about their history of systemic and ocular disease, history of ocular surgery, age, sex, and subjective visual disturbance during the day and night. The ocular problems of the participants were confirmed through slit-lamp examination, and only participants with no anterior surface problems were enrolled. This study was approved by the Institutional Review Board of Yonsei University Health System (IRB no. 1-2021-0052), and informed consent was waived due to the retrospective nature of the study. All methods were performed in accordance with the relevant guidelines and regulations.

### Measurements

Two CST devices, a manual CST (OPTEC-6500, Stereo Optical) and an automated CST (CGT-2000, Takagi Seiko Co., Ltd.), were used in this study. The manual CST was used in mesopic and photopic conditions with spatial frequencies of 1.5, 3, 6, 12, and 18 cycles per degree of visual angle (cpd) without glare. Monocular contrast sensitivity was measured at far (3 m) distance under photopic conditions at 85 cd/m^2^ and mesopic conditions at 3 cd/m^2^ with optimum refractive correction and a natural pupil, according to the manufacturer’s recommendation^18^.

The automated CST was also used for mesopic and photopic conditions with six target sizes (6.3°, 4.0°, 2.5°, 1.6°, 1.0°, and 0.64°) and 14 contrast levels (0.0071–0.64) without glare. Monocular contrast sensitivity was measured at far (5 m) distance under photopic at 100 cd/m^2^ and mesopic at 5 cd/m^2^ with optimum refractive correction and a natural pupil, according to the manufacturer’s recommendation^19^.

Two measurements were taken for each platform. Mesopic contrast sensitivity was tested first, and photopic contrast sensitivity was assessed after an interval of 30 min. The right eye was tested first in both mesopic and photopic contrast sensitivity. The CST device used first were randomly allocated. 48% (n = 20) of patients with history of refractive surgery and 50% (n = 20) of patients without history of refractive surgery were tested with manual CST first. Both CST were done on the same day with an interval of at least 1 h between the two sessions. The CST was repeated on another day with the same sequence.

All psychophysical tests of this study were performed in the same testing laboratory where standardized lighting conditions were ensured by blocking daylight.

The time taken to complete an examination was measured for both manual and automated CST. For manual CST, a stopwatch was started when the patient was shown the first row of targets and was stopped when the patient finished reading the last row of targets. For automated CST, a stopwatch was started as the start button was pressed and was stopped when the examination ended with a beep sound. After the examination, time taken for mesopic CST and photopic CST was added to calculate the time taken for each device. The time taken to explain the test method or register patient data to the device was not included in the examination time.

For consistency of data, all examinations were performed by a single physician.

### Statistical analysis

Descriptive statistics were used to characterize baseline characteristics and comorbidities. Continuous variables are expressed as mean ± standard deviation (SD), and categorical variables are reported as frequency (percentage).

To examine the test–retest reliability of each method and the correlations between the two methods, the intraclass correlation coefficient (ICC) was calculated. The ICC of two CST measurements was calculated under absolute-agreement, 2-way random-effects model to calculate the interrater reliability^20^. In concordance with a widely used guideline for reporting ICC by Koo et al.^20^, ICC values less than 0.5, between 0.5 and 0.75, between 0.75 and 0.9, and greater than 0.90 were interpreted as poor, moderate, good, and excellent reliability, respectively, based on the 95% confidence interval of the ICC estimate. Area under the log contrast sensitivity formula (AULCSF) was calculated for each examination using the methods described previously^21^. The difference between AULCSF in mesopic and photopic conditions was used to compare the quality of vision in different settings as in previous studies^22,23^. The Bonferroni method was applied to approve the statistical significance.

All tests were two-tailed, with P < 0.05 considered statistically significant. Statistical analyses were conducted using SPSS (version 23.0; IBM, Armonk, NY) and MedCalc Statistical Software version 14.8.1 (MedCalc, Ostend, Belgium).

## Results

A total of 41 patients were analyzed in this study; 51.2% were female, and the mean age was 26.6 ± 3.7 years. In total, 164 measurements of 82 eyes (20 eyes with prior LASIK, 20 eyes with prior LASEK, and 42 eyes without a history of ocular surgery) were performed.

The baseline characteristics of patients with or without a history of refractive surgery are presented in Table 1. Those who received refractive surgery were older (28.1 ± 4.4 years vs. 25.3 ± 2.2 years), were more frequently female (60% vs. 43%) and reported more decreased subjective night vision (55% vs. 38%, P = 0.03) compared with those who did not receive refractive surgery.

**Table 1:** Baseline characteristics of patients based on history of refractive surgery.

Variables	Eyes with history of refractive surgery (n = 42)	Eyes without history of refractive surgery (n = 40)	*P*-value
Age, years (mean ± SD [range])	25.3 ± 2.2 [23–32]	28.1 ± 4.4 [23–39]	< 0.001*
Female (%)	18 (43%)	24 (60%)	0.028*
Number of patients who tested manual CST first (%)	20 (48%)	20 (50%)	0.84
Years since refractive surgery (mean ± SD [range])	–	5.5 ± 3.5 [1–12]	–
Subjective night vision decrease (%)	16(38%)	22(55%)	0.030*

*Indicates statistically significant results.

### Time to complete examinations

The mean time taken to complete one full examination (including four steps; both right and left eyes in mesopic and photopic conditions) was significantly shorter in automated CST examinations compared with manual CST examinations (396.4 ± 20.4 s vs. 286.8 ± 2.3 s, respectively; P < 0.001) (Table 2). Examination time was significantly shorter in the second examination than in the first examination using the manual CST (P = 0.01); however, the first examinations performed with the automated CST were even quicker than the second examinations taken with the manual CST (P < 0.001).

**Table 2:** Mean time taken to complete one full examination using manual and automated CST.

Examination time	Manual CST	Automated CST	*P-*value
First examination (s)	431.1 ± 21.8	285.2 ± 4.1	< 0.001*
Second examination (s)	361.8 ± 14.6	288.5 ± 2.0	< 0.001*
*P-*value	0.003*	0.51	

*Indicates statistically significant results.

### Test repeatability

The test–retest ICC analysis of both CST devices revealed that the manual CST showed good repeatability (i.e., ICC value of 0.75–0.90 based on the 95% confidence interval^20^) in both photopic and mesopic conditions, while the automated CST showed moderate repeatability (i.e., ICC value of 0.50–0.90 based on the 95% confidence interval^20^) in both mesopic and photopic conditions (Appendix 1 in the Supplementary material). The ICC of the inter-test analysis was moderate in both photopic and mesopic conditions (Appendix 1 in the Supplementary material).

### Ceiling and floor effects

Appendix 2 and 3 in the Supplementary material show the proportion of eyes with a maximum or minimum score in examinations using the manual and automated CSTs according to the history of refractive surgery. The ceiling effect (i.e., obtaining the maximum score on the chart) and floor effect (i.e., obtaining the minimum score on the chart) were more prominent with the manual CST than with the automated CST in both patients with and without refractive surgery. There was also a floor effect in eyes with and without refractive surgery at the highest spatial frequency in automated CST examinations. Meanwhile, the floor effect in manual CST examinations occurred at both the highest and second-highest spatial frequencies in both patients with and without refractive surgery.

### Correlation with history of refractive surgery

**Figure 2:**
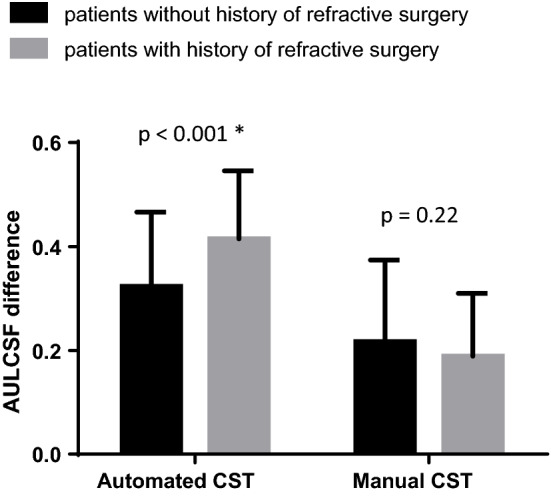
AULCSF difference of photopic and mesopic conditions in automated and manual CST devices according to history of refractive surgery. AULCSF, area under log contrast sensitivity function; CST, contrast sensitivity test. *Statistically significant results.

There was no significant difference between the AULCSF of patients who did or did not undergo refractive surgery in either examination (Table 3). However, patients who underwent refractive surgery showed significantly larger AULCSF difference (calculated as photopic AULCSF − mesopic AULCSF) in automated CST examinations compared with patients without a history of ocular surgery (AULCSF difference 0.415 vs. 0.323 in patients with and without refractive surgery, respectively; P < 0.001). However, there was no significant difference between patients with and without refractive surgery in manual CST examinations (AULCSF difference 0.189 vs. 0.217 in patients with and without refractive surgery, P = 0.22) (Fig. 2).

**Table 3:** Comparison of mesopic AULCSF, photopic AULCSF, AULCSF difference in patients with or without history of refractive surgery.

	Manual CST			Automated CST		
	Eyes without refractive surgery (n = 84)	Eyes with prior refractive surgery (n = 80)	P-value	Eyes without refractive surgery (n = 84)	Eyes with prior refractive surgery (n = 80)	P-value
Mesopic AULCSF	1.81 ± 0.23	1.79 ± 0.21	0.79	1.30 ± 0.22	1.26 ± 0.20	0.29
Photopic AULCSF	1.59 ± 0.30	1.60 ± 0.26	0.66	1.62 ± 0.20	1.68 ± 0.18	0.05
AULCSF difference	0.22 ± 0.16	0.19 ± 0.12	0.22	0.32 ± 0.14	0.42 ± 0.13	< 0.001*

AULCSF difference was calculated as Photopic AULCSF − Mesopic AULCSF.

AULCSF: area under log contrast sensitivity function; CST: contrast sensitivity test.

*Indicates statistically significant results.

### Correlation with decreased subjective night vision

**Figure 3:**
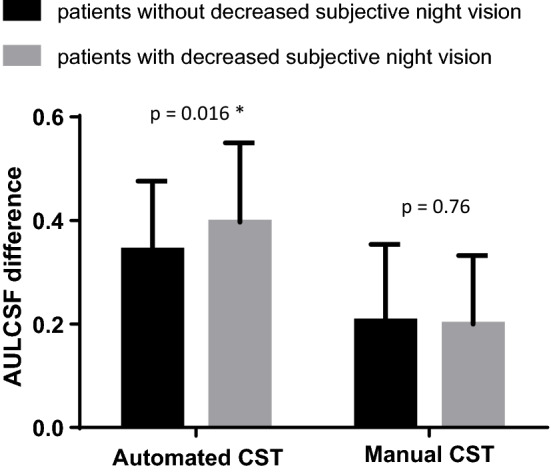
AULCSF difference of photopic and mesopic conditions in automated and manual CST devices according to decreased subjective night vision. AULCSF, area under log contrast sensitivity function; CST, contrast sensitivity test. *Statistically significant results.

Patients who reported decreased subjective night vision showed significantly larger AULCSF differences in automated CST examinations compared with those who did not report decreased subjective night vision (AULCSF difference 0.397 vs. 0.343 in patients who did and did not report decreased subjective night vision, respectively; P = 0.02). However, there was no significant difference between patients who did and did not report decreased subjective night vision in manual CST examinations (AULCSF difference 0.200 vs. 0.206 in patients who did and did not report decreased subjective night vision, respectively, P = 0.76) (Fig. 3).

## Discussion

The present study revealed the following findings. First, in the ICC analysis, the manual CST performed better than the automated CST, but both showed moderate to good test repeatability and fair to good inter-test correlation. In addition, the ceiling and floor effects were both more prominent with the manual CST than with the automated CST in both patients with and without refractive surgery. These results show that the automated CST shows comparable repeatability and reliability compared to manual CST devices.

Second, the mean time taken to complete one full examination was significantly shorter when using the automated CST compared with the manual CST. Contrast sensitivity relates the visibility of a spatial pattern to both its size and contrast and is therefore a more comprehensive assessment of visual function than visual acuity but can be more time-consuming^24^. Therefore, efficiency has become an important part of CST as well as precision, and there have been many approaches reduce time to detect contrast sensitivity changes^24,25^. In this study, the mean time taken to complete one full examination was significantly shorter when using the automated CST compared with the manual CST. Unlike the manual CST, in which patients are given unlimited patient response time, the automated CST requires a timed response, which not only reduce the overall time taken for each test, but also reduce the bias due to exposure time to visual stimuli. Human vision requires certain contrast, size, and exposure time for an object to be detected^26,27^. Therefore, the time of exposure to a stimulus is a critical variable in assessing the patient’s contrast sensitivity, while the manual CST lose examining value because the time of the motor response is not controlled^27^.

Finally, patients who underwent refractive surgery showed significantly larger AULCSF differences in automated CST examinations compared with patients without a history of ocular surgery, while there was no significant difference in manual CST examinations. Patients who reported decreased subjective night vision showed significantly larger AULCSF differences in automated CST examinations compared with those who did not report decreased subjective night vision, while there was no significant difference in manual CST examinations. These results imply that compared with manual CST, the automated CST correlated well with subjective night vision decrease and had higher sensitivity to history of refractive surgery.

This superiority is probably due to improvements in the methodology of the automated CST. First, previous CST devices made use of vertical linear gratings that were biased for with-the-rule astigmatism and horizontal coma. Astigmatism and higher-order aberrations (coma, trefoil, and tetrafoil) cause lines to appear darker (higher contrast) in one angular orientation than in the orthogonal orientation. In contrast, the automated CST device made use of bull’s-eye sine-wave gratings, which is a rotationally symmetric target. For two patients with the same magnitude of astigmatism but at different orientations, the target will appear the same, and no advantage or disadvantage will happen based on the orientation of the appearance of the target^28^. Second, the automated CST allows a blanking period between the presentation of each stimulus, allowing for a more accurate test. Third, while the manual CST have a high probability of correct guessing with only 3 answer choices, and is more prone to false positives, the automated CST system reduced false positivity by randomly presenting the lowest contrast target^17^.

Most previous studies performed CSTs using only the manual CST vision testing system, with a background luminance of 3 cd/m^2^ for mesopic conditions and 85 cd/m^2^ for photopic conditions, while automated CST was performed under a background luminance of 5 cd/m^2^ for mesopic and 100 cd/m^2^ for photopic conditions, which may have resulted in more prominent differences between photopic and mesopic conditions. In addition, the manual CST is based on spatial frequencies of 1.5, 3, 6, 12, and 18 cpd, while the automated CST is conducted under visual angles of 6.3°, 4°, 2.5°, 1.6°, 1°, and 0.64°, deviating the test results to lower spatial frequency, equal to a larger visual angle^29^.

To our knowledge, no previous studies have compared the reproducibility and correlation of the manual and automated CSTs. While the manual CST is typically criticized for being prone to guessing and showing low repeatability under mesopic conditions^30^, the automated CST is typically criticized for poor repeatability and ceiling effects in normal individuals^31^. In this study, both showed moderate to good test repeatability and moderate inter-test correlation. In addition, compared with a manual CST, the automated CST showed 33.8% and 20.2% decreases in time in the first and second examinations, respectively. The ceiling and floor effects were also smaller with the automated CST. These findings suggest that an automated CST can be a good alternative to manual CST in young adults with normal visual acuity.

The current literature is unclear regarding the long-term compromise of contrast sensitivity after refractive surgery^32^. Some demonstrate only a temporary decrease in contrast sensitivity within a few months after refractive surgery^33,34^, while others demonstrate a persistent decrease in contrast sensitivity, especially in mesopic conditions and in intermediate to low cycles per degree^13,35,36^. Increased ocular higher-order aberrations and the oblate shape of the cornea following refractive surgery are suggested to be the reason for compromised contrast sensitivity^13,37,38^.

The present study reveals the long-term outcome in contrast sensitivity of patients who underwent refractive surgery at least one year earlier (mean time 5.5 ± 3.5 [range 1–12] years). Eyes with prior LASIK or LASEK had similar contrast sensitivity to normal eyes in both photopic and mesopic conditions (all P > 0.05). However, the AULCSF difference was significantly higher in eyes with prior refractive surgery in automated CST examinations, while no significant difference was seen in manual CST examinations. In addition, patients who reported decreased subjective night vision showed significantly larger AULCSF differences in automated CST examinations compared with those who did not report decreased subjective night vision, while there was no significant difference in manual CST examinations. In this study, the automated CST showed higher sensitivity to the presence of a history of refractive surgery and had better correlations with subjective night vision decrease compared with manual CST.

This study has several limitations. First, this retrospective study with a relatively small sample size is prone to higher variability and selection bias. Second, the patients enrolled in this study underwent refractive surgery from different hospitals, and the modality of refractive surgeries performed on each patient will vary. Finally, the effects of presbyopia were not determined. However, the age at onset of 40 years for presbyopia was widely used for Asian populations in previous epidemiologic studies^39,40^, and only subjects aged < 40 years were analyzed in this study. Another study reported the overall prevalence of functional presbyopia to be only 9.07% in Asian subjects aged 35–44 years^41^.

Despite these limitations, this study presents the first comparison of two contrast sensitivity devices that are widely used in the real world.

Compared with a manual CST, the automated CST took less time to complete and correlated well with subjective night vision decrease in patients after refractive surgery. These findings suggest that an automated CST can be a good alternative to a manual CST, with limited repeatability but higher sensitivity to the presence of a history of refractive surgery and with better correlations with subjective night vision decrease.

## Supplementary Information

Supplementary Information.

## Data Availability

Restrictions apply to the availability of these data, since they contain information that could compromise the patients’ privacy, and so are not publicly available. Data are however available upon reasonable request upon the corresponding author and with permission of Yonsei University College of Medicine.
