# Supplementary material for: Comparison of two different contrast sensitivity devices in young adults with normal visual acuity with or without refractive surgery
Source: Sci Rep. 2022 Jul 28;12:12882. doi: 10.1038/s41598-022-16855-3 (PMC9334259; doi:10.1038/s41598-022-16855-3)
Supplement: Supplementary file 1 — Supplementary Information. [file 41598_2022_16855_MOESM1_ESM.docx]

**Supplementary Material**

**Comparison of two different contrast sensitivity devices in young adults with normal visual acuity with or without refractive surgery**

Hyunjean Jung, MD,*^1^ Sung Uk Han, MD, ^*1^ Sangyeop Kim, MD, ^1^ Hyunmin Ahn, MD, ^1^ Ikhyun Jun, MD, PhD, ^1,2^ Hyung Keun Lee, MD, PhD,^1^ Kyoung Yul Seo, MD, PhD, ^1,2^ and Tae-im Kim, MD, PhD ^†1,2^

^1^ Institute of Vision Research, Department of Ophthalmology, Yonsei University College of Medicine, Seoul, South Korea

^2^ Corneal Dystrophy Research Institute, Department of Ophthalmology, Severance Hospital, Yonsei university, Seoul, Republic of Korea.

[*The first two authors contributed equally to this work]

[†Corresponding Author]

**^†^Corresponding author:**

Tae-im Kim, MD, PhD

50-1 Yonsei-ro, Seodaemun-gu, Seoul 03722, Republic of Korea

Phone: +82-2-2228-3570

Fax: +82-2-312-0541

E-mail: [TIKIM@YUHS.AC](mailto:TIKIM@YUHS.AC)

Appendix 1. Intraclass coefficient for the test-retest reliability of contrast sensitivity tests using manual and automated CST

| Manual CST | | | Automated CST | | | Inter-test analysis | | |
| --- | --- | --- | --- | --- | --- | --- | --- | --- |
|  | ICC | 95% CI |  | ICC | 95% CI |  | ICC | 95% CI |
| Mesopic | 0.86 | 0.78-0.91 | Mesopic | 0.84 | 0.74-0.89 | Mesopic | 0.71 | 0.61-0.79 |
| Photopic | 0.88 | 0.82-0.92 | Photopic | 0.80 | 0.69-0.87 | Photopic | 0.63 | 0.50-0.73 |

CST; contrast sensitivity test.

ICC; Intraclass coefficient.

Appendix 2. Proportion of maximum and minimum scores in Manual CST according to history of refractive surgery

|  | No. reaching maximal score (%) | | No. reaching minimal score(%) | | |
| --- | --- | --- | --- | --- | --- |
| Spatial frequency (cpd) | Eyes with history of refractive surgery  (n=168) | Eyes without history of refractive surgery  (n=160) | | Eyes with history of refractive surgery  (n=168) | Eyes without history of refractive surgery  (n=160) |
| 1.5 | 11 (6.5%) | 9 (5.6%) | | 0 | 0 |
| 3.0 | 4 (2.4%) | 3 (1.7%) | | 0 | 0 |
| 6.0 | 5 (3.0%) | 15 (9.4%) | | 0 | 0 |
| 12.0 | 3 (1.8%) | 6 (3.8%) | | 12(7.1%) | 7 (4.4%) |
| 18.0 | 3 (1.8%) | 2 (1.3%) | | 19(11.3%) | 17 (10.6%) |

CST; contrast sensitivity test.

Appendix 3. Proportion of maximum and minimum scores in Automated CST according to history of refractive surgery

|  | Maximal score (%) | | Minimal score(%) | | |
| --- | --- | --- | --- | --- | --- |
| Visual angle | Eyes with history of refractive surgery  (n=168) | Eyes without history of refractive surgery  (n=160) | | Eyes with history of refractive surgery  (n=168) | Eyes without history of refractive surgery  (n=160) |
| 6.3⁰ | 2 (1.2%) | 1 (0.6%) | | 0 | 0 |
| 4.0⁰ | 3 (1.8%) | 6 (3.8%) | | 0 | 0 |
| 2.5⁰ | 2 (1.2%) | 2 (1.3%) | | 0 | 0 |
| 1.6⁰ | 1 (0.6%) | 1 (0.6%) | | 0 | 0 |
| 1.0⁰ | 0 | 0 | | 0 | 0 |
| 0.64⁰ | 0 | 0 | | 11 (6.5%) | 7 (4.4%) |

CST; contrast sensitivity test.
